# Supplementary material for: Macrolides Decrease the Proinflammatory Activity of Macrolide-Resistant Streptococcus pneumoniae
Source: Microbiol Spectr. 2023 May 16;11(3):e00148-23. doi: 10.1128/spectrum.00148-23 (PMC10269745; doi:10.1128/spectrum.00148-23)
Supplement: Supplemental file 1 — Supplemental material. Download spectrum.00148-23-s0001.pdf, PDF file, 0.2 MB [file spectrum.00148-23-s0001.pdf]

Supplemental Material

Figure S1

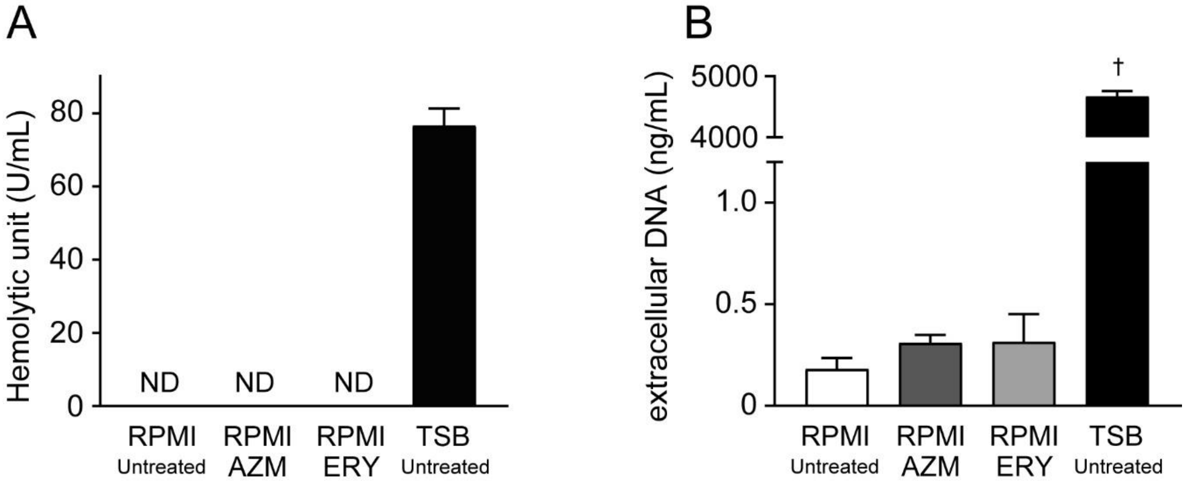

Supplemental Figure S1. RPMI 1640 medium seldom induces autolysis and subsequent release of extracellular DNA in *S. pneumoniae* strain NU4471, leading to the impaired hemolytic activity of pneumococcal culture supernatants.

Macrolide-resistant *S. pneumoniae* strain NU4471 was grown in a tryptic soy broth or RPMI 1640 medium supplemented with 10% fetal bovine serum in the presence or absence of 5 μg/mL azithromycin (AZM) or erythromycin (ERY) until bacterial growth reached the stationary phase (OD_{600} = 0.55). The pneumococcal culture supernatants were obtained after centrifugation, as described in Materials and Methods. (A) The hemolytic activity of each cell-free supernatant was determined using a 1% suspension of sheep erythrocytes (Nippon Bio-Test Laboratories, Tokyo, Japan). (B) To determine the concentration of pneumococcal extracellular DNA released through autolysis in the culture supernatant, absolute

quantification was performed using real-time PCR. Primers were designed to detect the pneumolysin-encoding genes. The forward primer sequence was 5'-AGCGATAGCTTTCTCCAAGTGG-3′ and the reverse primer sequence was 5′-CTTAGCCAACAAATCGTTTACCG-3′. Data represented the mean ± SD of quadruplicate experiments and were evaluated using a one-way analysis of variance with Tukey's multiple comparisons test. † indicates significant difference compared to the RPMI 1640 medium-based supernatant from the macrolide-untreated control group at $P < 0.05$.

Figure S2

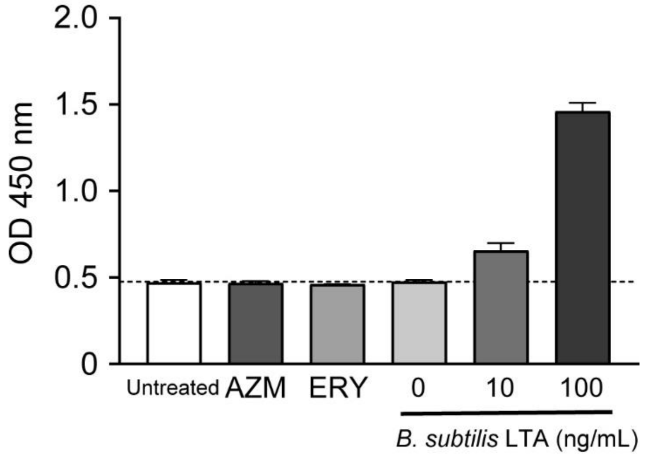

Supplemental Figure S2. Pneumococcal lipoteichoic acid cannot be detected using a commercial LTA ELISA kit.

Macrolide-resistant *S. pneumoniae* strain NU4471 was grown in RPMI 1640 medium supplemented with 10% fetal bovine serum in the presence or absence of 5 μg/mL azithromycin (AZM) or erythromycin (ERY) until bacterial growth reached the stationary phase (OD_{600} = 0.55). Pneumococcal culture supernatants were obtained after centrifugation, as described in Materials and Methods. Lipoteichoic acid (LTA) ELISA kit (Cell Biolabs Inc., San Diego, CA, USA) was used to determine LTA levels in the pneumococcal supernatants following the manufacturer's instructions. LTA from *Bacillus subtilis* (Cell Biolabs Inc.) was used as a positive control.
